# Genomic characterization of ribitol teichoic acid synthesis in *Staphylococcus aureus*: genes, genomic organization and gene duplication

**DOI:** 10.1186/1471-2164-7-74

**Published:** 2006-04-05

**Authors:** Ziliang Qian, Yanbin Yin, Yong Zhang, Lingyi Lu, Yixue Li, Ying Jiang

**Affiliations:** 1 Molecular and Investigative Toxicology, Merck Research Laboratories, WP45-330, West Point, PA 19486, USA; 2 College of Life Sciences, National Laboratory of Genetic Engineering and Protein Engineering, Center of Bioinformatics, Peking University, Beijing 100871, China; 3 Bioinformatics Center, Shanghai Institutes for Biological Sciences, Chinese Academy of Sciences, 320 Yueyang Road, Shanghai 200031, China; 4 Graduate School of the Chinese Academy of Sciences, 19 Yuquan Road, Beijing 100039, China

## Abstract

**Background:**

*Staphylococcus aureus *or MRSA (Methicillin Resistant *S. aureus*), is an acquired pathogen and the primary cause of nosocomial infections worldwide. In *S. aureus*, teichoic acid is an essential component of the cell wall, and its biosynthesis is not yet well characterized. Studies in *Bacillus subtilis *have discovered two different pathways of teichoic acid biosynthesis, in two strains W23 and 168 respectively, namely teichoic acid ribitol (*tar*) and teichoic acid glycerol (*tag*). The genes involved in these two pathways are also characterized, *tarA*, *tarB*, *tarD*, *tarI*, *tarJ*, *tarK*, *tarL *for the *tar *pathway, and *tagA*, *tagB*, *tagD*, *tagE*, *tagF *for the *tag *pathway. With the genome sequences of several MRSA strains: Mu50, MW2, N315, MRSA252, COL as well as methicillin susceptible strain MSSA476 available, a comparative genomic analysis was performed to characterize teichoic acid biosynthesis in these *S. aureus *strains.

**Results:**

We identified all *S. aureus tar *and *tag *gene orthologs in the selected *S. aureus *strains which would contribute to teichoic acids sythesis.Based on our identification of genes orthologous to *tarI, tarJ*, *tarL*, which are specific to *tar *pathway in *B. subtilis *W23, we also concluded that *tar is *the major teichoic acid biogenesis pathway in *S. aureus*. Further analyses indicated that the *S. aureus tar *genes, different from the divergon organization in *B. subtilis*, are organized into several clusters in cis. Most interesting, compared with genes in *B. subtilis tar *pathway, the *S. aureus *tar specific genes (*tarI,J,L*) are duplicated in all six *S. aureus *genomes.

**Conclusion:**

In the *S. aureus *strains we analyzed, *tar *(teichoic acid ribitol) is the main teichoic acid biogenesis pathway. The *tar *genes are organized into several genomic groups in cis and the genes specific to *tar *(relative to *tag*): *tarI*, *tarJ*, *tarL *are duplicated. The genomic organization of the *S. aureus tar *pathway suggests their regulations are different when compared to *B. subtilis tar *or *tag *pathway, which are grouped in two operons in a divergon structure.

## Background

*Staphylococcus*. Aureus (*S. aureus*) is a Gram-positive bacterium, which causes a variety of suppurative infections and toxinoses in humans. The death rate associated with *S. aureus *infection is still high even with antimicrobial drug treatments due to the development of antibiotic resistance in Methicillin Resistant *Staphylococcus Aureus *(MRSA) strains. Current developments in antimicrobial therapeutics show little efficacy in treating *S. aureus *and this bacterium remains a major human health threat. *S. aureus*, and in particular its cell wall, remain a major target of glycopeptide antibiotics and focus of bacteriology research.

Teichoic acids, polymers of alternating phosphate and alditol groups, in addition to peptidoglycan are an essential component of bacterial cell walls. Teichoic acid biosynthesis in *S. aureus *has not been well characterized. *B. subtilis *and *S. aureus *are both phylogenetically classified into *Bacillus/Staphylococcus *group. Unlike that in *S. aureus*, the teichoic acid biogenesis in *B. subtilis *is well understood[1,2]. There are two major types of cell wall teichoic acid in *B. subtilis*, poly(glycerol phosphate) [poly(GroP)] in strain 168 [2] and poly(ribitol phosphate) [poly(RboP)] in strain W23 [3]. In strain 168, the biosynthesis of teichoic acid poly(GroP) involves genes *tagA, tagB, tagD, tagE, and tagF*. These *tag *genes are organized in a divergon of two divergently transcribed operons, *tagAB *and *tagDEF*[4,5]. In W23, the biosynthesis of teichoic acids poly(RboP) involves genes *tarA, tarB, tarD, tarF, tarI, tarJ, tarK and tarL*. The *tar *genes, similar to the *tag *genes, are also organized in a divergon, *tagAB-tagDEF *[2]. The biogenesis of essential wall teichoic acid in *B. subtilis *differs in these two strains, yet still shares similar enzymatic steps. Functions of *tagB, tagD, tagF *were identified biochemically [1,6]. Based on high sequence similarity, *tarA, tarB, tarD*, and *tarF *are believed to perform similar enzymatic reactions as *tagA*, *tagB*, *tagD*, and *tagF *do respectively [2], while *tarI, tarJ, tarK and tarL *carry functions specific to poly(RboP) teichoic acid biosynthesis [2].

It has been reported that *S. aureus *H contains ribitol in its cell wall as does *B. subtilis *W23 [7,8]. It has also been shown that a Tar*D *like enzyme exists in *S. aureus *with catalytic characteristics different from *B. subtilis *Tag*D *[9]. These studies suggest poly(RboP) could be one of the cell wall teichoic acids in some *S. aureus *strains, yet this has not been unequivocally demonstrated.

Recently the genomes of MRSA strains Mu50, MW2, N315, MRSA252, and COL as well as methicillin susceptible strain MSSA476 have been sequenced [9-12]. The available sequence information enables us to take a comparative genomics approach to study the genomic requirements of wall teichoic acid in *S. aureus *by comparing them to the genes involved in *B. subtilis *teichoic acid synthesis.

We took all *B. subtilis tar *and *tag *genes, computationally to identify all orthologous genes supposedly involved in wall teichoic acid biogenesis in the *S. aureus *strains Mu50, MW2, N315, MRSA252, MSSA476 and COL. Our results suggest that poly(RboP), rather than poly(GroP), is the major teichoic acid in these strains. We also report the genomic organization of the teichoic acid biogenesis genes, which is different from the divergon organization in *B. subtilis *W23 and 168.

## Results

### Identify genes involved in wall teichoic acid synthesis in *S. aureus *through comparative genomics analysis

**Figure 1:**
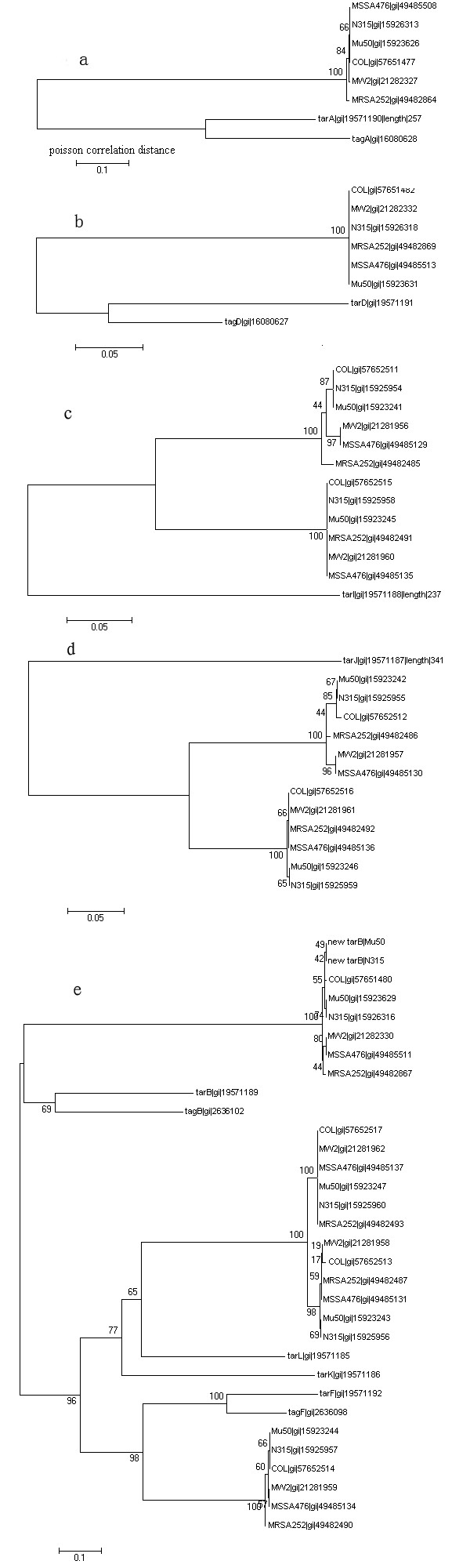
**Phylogenetic analysis to identify Tar/Tag orthologs in *S. aureus***. The phylogenetic tree shows the orthologs of *B. subtilis *Tar/Tag ORFs in *S. aureus *strains Mu50, MW2, N315, MRSA252, MSSA476 and COL. BLAST hits (statistically significant) were used as input. MEGA3 program was used to perform this analysis. NJ trees were constructed using PC (Poisson Correlation) distance and a bootstrap value of 500. After deleting distant branches (homologs but not orthologs), final tree generated. Figure 1a depicts Tar/TagA orthologs. Figure 1b depicts Tar/TagD orthologs. Figure 1c depicts double TarI orthologs in each *S. aureus *strain. Figure 1d depicts double TarJ orthologs in each *S. aureus *strain. Figure 1e depicts orthologs of Tar/TagB, Tar/TagF, TarL and once again, two orthologs of TarL in each *S. aureus *strain are found. However, there is no ortholog of TarK.

In order to identify the genes concerned with wall teichoic acids synthesis in *S. aureus*, strains Mu50, MW2, N315, MRSA252, MSSA476 and COL, amino acid sequences of all *tar *genes in *B. subtilis *W23 strain and *tag *genes in *B. subtilis *168 strain from GenBank were BLASTed against the Refseq ORFs of Mu50, MW2, N315, MRSA252, MSSA476 and COL. Significant hits were identified (data not shown), and further subjected to phylogenetic analysis. The analysis led to the identification of the corresponding *tar *or *tag *orthologs in the *S. aureus *strains we examined. "Ortholog" here is technically defined as those with the best phylogenetic similarity. By deleting branches of less homologous hits, the trees in Figure 1 were generated, indicating the orthologous *tar *or *tag *genes in those *S. aureus *strains. *tarB*, *tarF*, *tarL*, *tarK *from *B. subtilis *W23 and *tagB*, *tagF *from *B. subtilis *168 share common BLAST hits in these *S. aureus *strains. We grouped these genes as well as their BLAST hits together to perform the phylogenetic analysis and built an integral tree (Figure 1e) to show their respective orthologous relationship.

**Figure 2:**
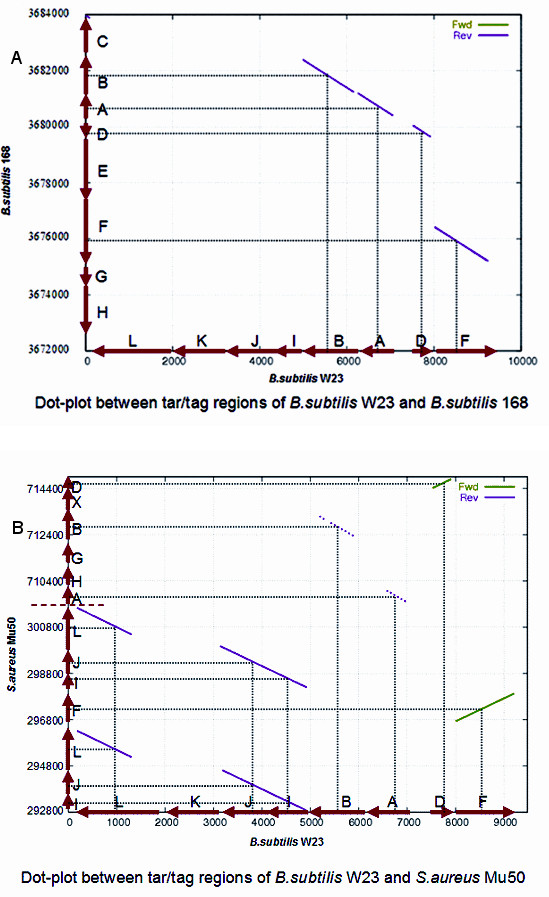
**Dot-blot analysis of tar/tag regions**. Homologous relationships are confirmed by dot-blot analyis. Figure 2a shows the synteny and the homologous genes in the W23 *tar *and 168 *tag *regions. Figure 2b shows the synteny and the orthologous relationships of genes involved teichoic acid synthesis in *B. subtilis *W23 and Mu50. In Mu50, the *tar *genes are not clustered together. We artificially brought them together to make the dot-plot easy to read. Strains MW2, N315, MRSA252, MSSA476 and COL are the same as Mu50 (not shown).

Interestingly, these *S. aureus *orthologs, identified by phylogenetic analysis, shown in Figure 1, are actually the best BLAST hits (not shown). (We subsequently performed a "reverse BLAST hit" analysis on the identified *S. aureus *ORFs.) We took the *S. aureus *ORFs (the best BLAST hits) and BLASTed them back against *B. subtilis *strain 168 ORFs. Their corresponding *tag *genes were also identified as the best BLAST hits (not shown). The reverse BLAST was not performed for W23 strain since its genome sequence is not currently available. The reverse best BLAST hit analysis was in agreement with phylogenetic clustering in identifying the orthologous genes involved in wall teichoic acid synthesis in examined *S. aureus *strains (Figure 1). The orthologous relationship is also summarized in Table 1. We further performed dot-blot analysis, which also support the identified corresponding orthologous relationships (Figure 2a, 2b).

### *S. aureus *contains *tarF *instead of *tagF*

**Figure 3:**
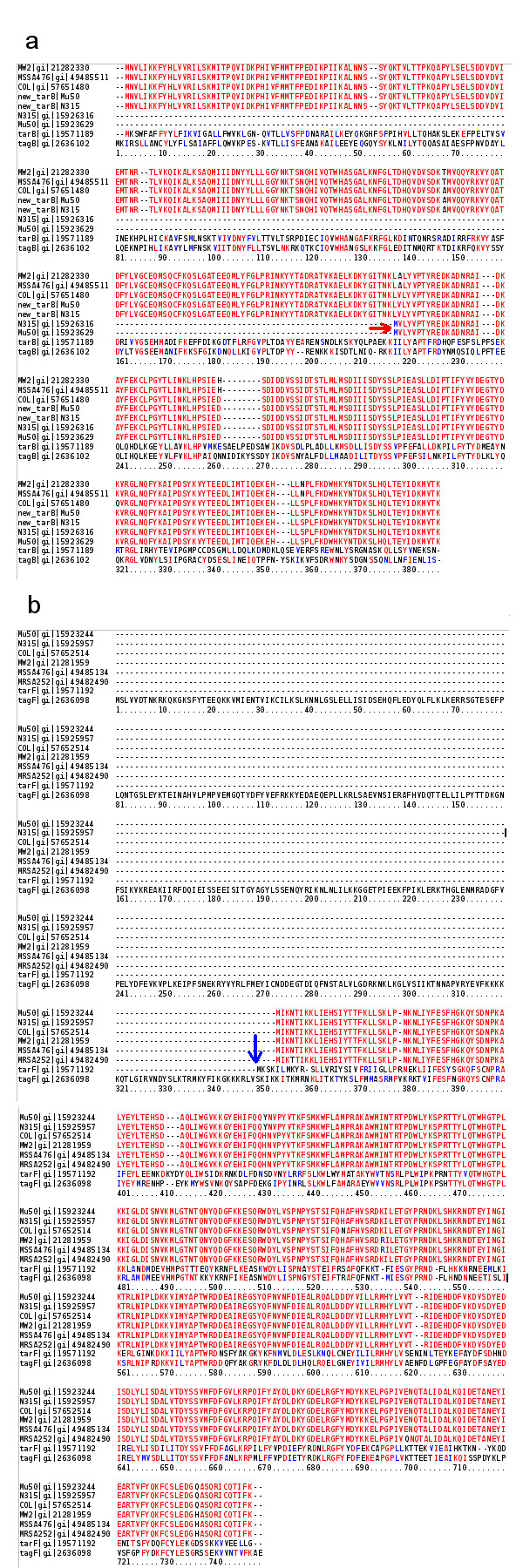
**Multiple sequence alignment of TarB/TagB, TarF/TagF (protein) and their orthologs**. Figure 3a. TarB/TagB orthologous ORFs in Mu50 and N315 (Mu50|gi|15923269 and N315|gi|1592316) are much shorter than in other strains (red arrow), which is suspected as an annotation error. So ORF finder program was rerun on this region and new_tarBs in Mu50 and N315 were identified. As shown in figure, new_tarB|Mu50 is a upstream extension of Mu50|gi|15923269. new_tarB|N315 is a upstream extension of N315|gi|1592316. See Additional file 1 for sequences of new_tarBs. Figure 3b. The size and homology clear shows that the analyzed S. aureus strains contain TarF like protein rather than TagF (blue arrow).

The protein alignment of *S. aureus tagF/tarF *orthologs with *B. subtilis *TagF and TarF (Figure 3b) shows that *tagF/tarF *orthologs in these *S. aureus *strains are fully aligned with the W23 TarF in length, and also like *W23 *TarF, are about only half the size of 168 TagF. And only the C-terminal part of the larger TagF is significantly homologous to TarF[2].

In *B. subtilis*, despite sharing 60% identity, the size difference between TarF and TagF implies a functional difference. TagF and TarF both use CDP-glycerol as a substrate but do not carry out identical functions. In strain W23, TarF is likely to be responsible for the addition of the second glycerol-phosphate which completes the linkage unit process. While in 168, TagF polymerizes the complete glycerol-phosphate chain onto the first residue [6], which requires a bigger protein. Thus the size difference correlates with their functional difference between TagF and TarF. The size difference between TagF and TarF may also be used to differentiate *tar *or *tag *pathway in teichoic acid synthesis [5].

In the analyzed *S. aureus *strains, the size of *tagF/tarF *orthologs suggests the existence of TarF like function rather than TagF like function, and additional enzymes are required to complete teichoic acid synthesis[2].

### *S. aureus *utilize *tar *pathway instead of *tag *pathway for teichoic acid biosynthesis

In *B. subtilis *strain W23, *tarI, tarJ. tarK *and *tarL *are specific to *tar *pathway, which are responsible for the synthesis of RboP and the addition of poly(RboP) to the linkage unit [2] and are absent in strain 168's *tag *pathway. The identification of *tarI*, *tarJ *and *tarL *orthologs (Figure 1 and Table 1) and the *tarF *kind of function (instead of that of *tagF*) strongly suggest that the *tar *rather than the *tag *pathway is employed for cell wall teichoic acid synthesis in analyzed *S. aureus *strains. In the rest of the paper, these *S. aureus *wall teichoic acid synthesis genes are all referred as *tar *genes (Tar prefix will be used to refer to their protein products).

There are two copies for each *tar J*, *tarI *and *tarL *gene in those *S. aureus *strains (Figure 1, Table 1 and Figure 2). Interestingly, the *tarK *ortholog is missing from the analyzed *S. aureus *strains. In W23, *tarK *and *tarL *are identified to catalyze a similar function, but *tarL *could take a bigger substrate enzymatically (poly(Rbop)) [6]. Thus the absence of *tarK *ortholog in *S. aureus *could either mean it is functionally replaced by one of the two *tarL *or be compensated for by the extra copy of *tarL*.

### Identifying the correct full length *tarB *in *S. aureus *Mu50 and N315

According to multiple sequence alignment analysis and BLAST analysis of *B. subtilis *TarB/TagB (protein) with the corresponding *S. aureus *orthologs, the TarB ORFs in Mu50 and N315 were found to be notably shorter than *B. subtilis *TarB/TagB. They were also shown to lack half of the amino acid residues from the N-terminal of *B.subtilis *TarB/TagB (Figure 3a). One would expect that the translation start site would be further upstream in both strains. The ORF prediction was thus rerun on this genomic region (see methods), and the correct *tarB/tagB *in Mu50 and N315 were identified (see Additional file 1). The correct *tarB *in N315 is from 687949 to 689052, producing an ORF of 367 amino acids; the correct *tarB *In Mu50 is located between 712200 and 713303, also encoding an ORF of 367 amino acids. Both two new TarB ORFs were subsequently confirmed by a TBLASTN analysis against these two genomes with *B. subtilis *TarB as query. The new TarB ORFs in Mu50 and N315 strains and their alignment with the original incorrectly predicted ones are shown in Figure 3a.

### Genomic organization of *tar *genes and duplication of *tarI*, *tarJ *and *tarL *in *S. aureus *strains

**Figure 4:**
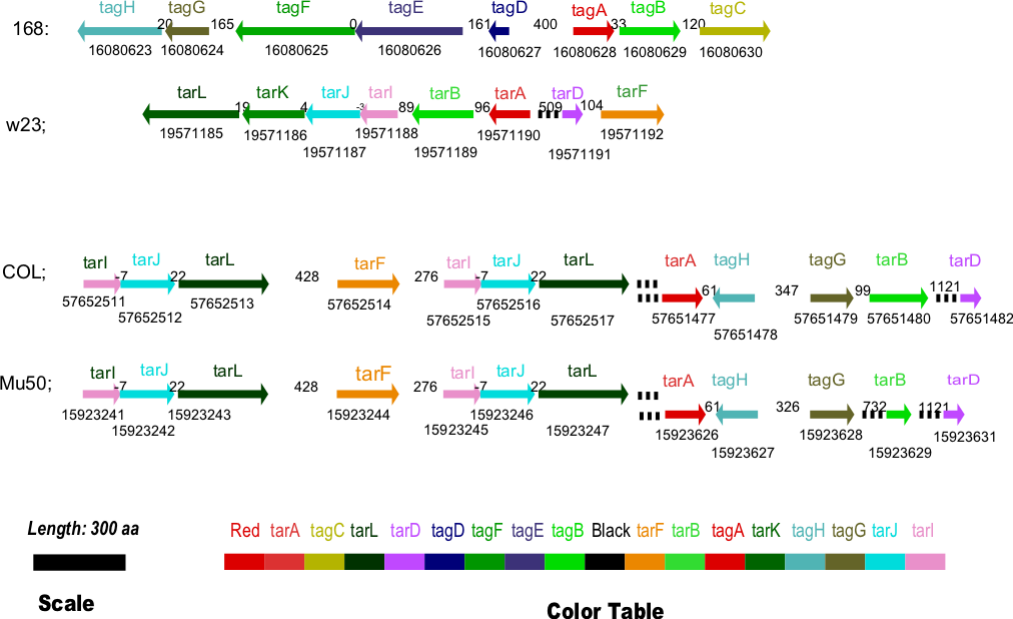
**Genomic organization of tar genes in *S. aureus***. Genomic organization of *tar/tag *genes W23, 168, *S. aureus *strains COL, Mu50 are shown here. The other 4 strains are not shown here since they have similar genomic organization (See Additional file 4 for complete figure). The top line of this figure shows the divergon organizations of *B. subtilis *W23 and 168 as a reference. The following two lines represented in arrows are graphic demonstration of genomic organization of the selected two *S. aureus *strains. Arrows in different colours represent different genes as illustrated by the colour table at the bottom. The length of each arrow is defined accurately by the scale, demonstrating the exact amino acid length of each gene. All six *S. aureus *strains share a similar genomic organization, which is quite different from *B. subtilis *W23 and 168 counterparts. GenBank gi numbers are indicated.

In *B. subtilis*, the wall teichoic acid synthesis genes are organized into a divergon, *tarABIJKL-tarDF *in W23 and *tagAB-tagDEF *in 168 (Figure 4, Additional file 4). However, in *S. aureus*, the *tar *genes seem are rather organized by genomic distance as *tarIJL-tarF-tarIJL-tarA-tarB-tarD *in cis orientation (Figure 4, see Additional file 4). This genomic organization is conserved in all six analyzed *S. aureus *strains.

**Figure 5:**
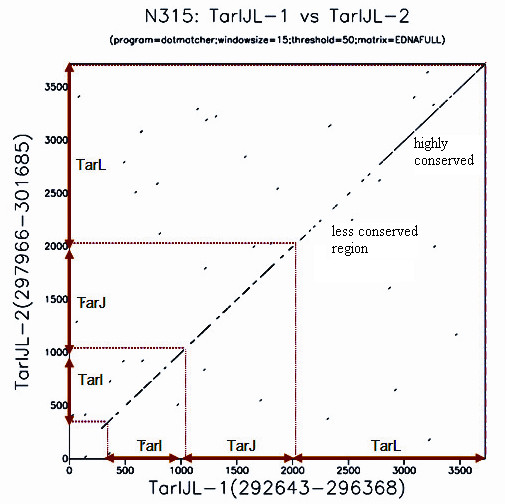
**Dot-blot analysis of the duplication of *tarIJL *in *S. aureus *N315**. The two *tarIJL *regions in Mu50 were aligned and dot-plot analysis was performed. The homology between these two regions is clearly shown. The high homology also indicates this duplication should not be a remote event. And part of *tarL *region is less conserved, which indicate that two *tarL *copies could have different functions. This phenomenon can be used to explain why there is no homologs of *tarK *in these *S. aureus *strains. Other *S. aureus *strains give similar results and are not shown here.

As shown above, BLASTP and phylogenetic analysis identify two copies of *tarI*, *tarJ *and *tarL *in each analyzed *S. aureus *strain, which are clustered into two *tarIJL *regions with the same gene order. Alignment and dot blot analysis of these two *tarIJL *regions in each *S. aureus *strain confirm this gene duplication (Figure 5, N315 is shown. The others with similar results are not shown). The relevant genomic sequences of *tarIJL *regions including the intergenic regions among I, J, K genes and the upstream 275 bp were used as an input for Dotmatcher program to perform dot-blot analysis of the two *tarIJL *regions (see Materials and Methods). We also ran the NCBI BLAST2seq program to align the two regions (not shown). These analyses further confirmed the homology between those two *tarIJL *regions, which strongly suggests the whole *tarIJL *region is duplicated. The high homology indicates the duplication should not be an evolutionary (or biologically) distal event. Why and how this gene duplication occurred is still a question that remains to be answered.

The dot blot analysis also demonstrated that a small section of the C-terminal of *tarJ *is not very conserved in the two copies of *tarJ*. The enzymatic implication of this is not yet clear. Similarly, the N-terminal of *tarL *(almost half the size of *tarL*) is neither homologous between the two *tarL *genes. It implies that one of the *tarL *is very likely to be the missing *tarK*.

## Discussion

To understand the biosynthesis of cell wall teichoic acid in *S. aureus *strains Mu50, MW2, N315, MRSA252, MSSA476 and COL, we took a bioinformatics approach to perform a comparative genomics analysis., We used the *B. subtilis *teichoic acid synthesis pathway as the base for comparison and identified all the genes essential to teichoic acid synthesis in these six *S. aureus *strains. Besides *tarA*/*tagA*, *tarB*/*tagB*, and *tarD*/*tagD *like genes, we identified *tarF *rather than *tagF *like gene and *tar *specific genes *tarI*, *tarJ *and *tarL*. The latter three ones are duplicated in these *S. aureus *strains.

In *B. subtilis*, *tarA*, *tarB*, *tarD *and *tarF *in W23 are the most similar to their counterparts in strain 168: *tagA*, *tagB*, *tagD *and *tagF *[6], whose functions in wall techoic acid synthesis are well understood [1,2]. Since *tarF *does not carry out a polymerization function as *tagF *does, W23 *tar *pathways requires *tarI*, *tarJ*, *tarK *and *tarL *to add poly(RboP) to the linkage unit to complete teichoic acid synthesis [2]. The identification of *tarF *like function and *tarI*, *tarJ*, *tarL *like genes strongly support the fact that the analyzed *S. aureus *strains use a *tar *like pathway and poly(RboP) is their major cell wall teichoic acids. This conclusion is consistent with the observations that a TarD rather than TagD like catalytic mechanism presentin *S. aureus *[13] and the identification of ribitol teichioc acid in the cell wall of *S. aureus *H [7,8].

**Figure 6:**
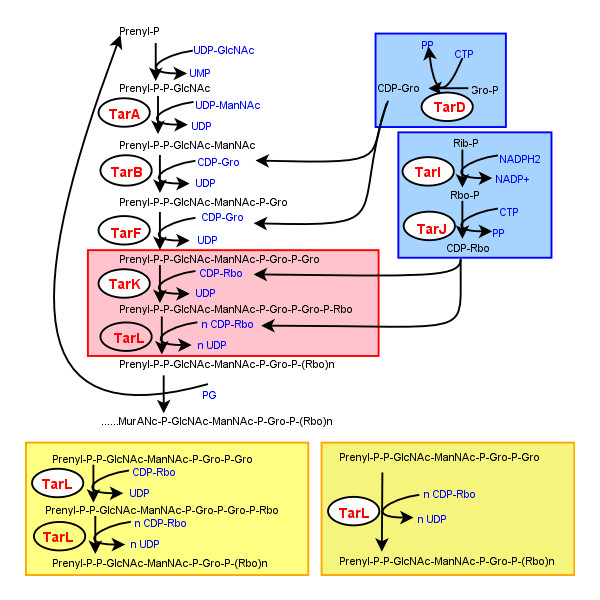
**The *tar *pathway in *S. aureus***. The top panel is the tar pathway in *B. subtilis *W23 [1], In *S. aureus *The TarK in B. subtilis is either replaced by TarL or merged with TarL as a one step (bottom blocks).

The *tarI*, *tarJ *and *tarL *in the six analyzed *S. aureus *strains are duplicated. Compared to the W23 *tar *pathway, *tarK *is absent in the six *S. aureus *strains. Based on the proposed function of *tarK *and *tarL *[2], we suggest that *tarK *is functionally redundant with one of the *tarL *in *S. aureus *or compensated by the duplication of *tarL*. Figure 6 schematically describes the tar pathway in the analyzed *S. aureus *strains. Compared to the proposed tar pathway in *B. subtilis *W23 (Figure 6, top panel) [1], the TarK in W23 could be replaced by one of the *S. aureus *TarL, or the TarK and TarL steps in W23 are actually merged as one TarL step in S. aureus (Figure 6, bottom blocks). Table 2 lists the putative enzymatic functions for *S. aureus *tar genes based on the SWISSPROT annotation of *B. subtilis *W23 tar genes. In this report, we also identified the correct ORFs for *tarB *in *S. aureus *strains N315 and Mu50, which are actually longer than the original ORFs in GenBank.

The genomic organizations of *tar *genes in *S. aureus *are quite different from *B. subtilis*. They are organized into several clusters in cis rather than the divergon in *B. subtilis*, and may be subjected to different regulatory mechanisms.

## Conclusion

As we analyzed, *tar *(teichoic acid ribitol) is the main teichoic acid biogenesis pathway in the *S. aureus *strains. And, the *tar *genes are organized into several genomic groups in cis and the genes specific to *tar *(relative to *tag*): *tarI*, *tarJ*, *tarL *are duplicated. The genomic organization of the *S. aureus tar *pathway suggests their regulations are different when compared to *B. subtilis tar *or *tag *pathway, which are grouped in two operons in a divergon structure.

## Methods

### Data sources

Six bacteria strains are included in this analysis: *Staphylococcus aureus *Mu50, MW2, N315, MRSA252, MSSA476, COL and *Bacillus subtilis *168. Their respective Refseq proteomes are downloaded from NCBI microbial genomes website [15]. The complete geonome sequence of *Bacillus subtilis *W23 is not available yet. The Tar proteins in W23 (A, B, D, F, I, J, K, L) are also downloaded from NCBI microbial genomes website [15].

### BLASTP search

The eight Tar proteins and five Tag proteins (A, B, D, E, F) were used as queries to BLASTP against the proteomes (ORFs) of *S. aureus *Mu50, MW2, N315, MRSA252, MSSA476 and COL. For each querying protein, using E value cut off of 1e-10, we collected all the significant hits.

To define the identified possible Tar/Tag homoglous proteins in six *S. aureus *strains, we also took the above identified *S. aureus *proteins (ORFs) to BLAST back against proteome of *B. subtilis*168. Mutual best BLAST hit is another evidence of the orthologous relationship.

### Multiple sequence alignment and phylogenetic tree building

The above selected BLAST hits from the combined dataset plus the relevant Tar protein are taken to do ClustalW alignment. The multiple sequence alignment result is further taken as input to do phylogenetic analysis.

Mega3 package is used for phylogenetic analysis and tree building. First the ClustalW produced .aln files were transformed into Mega readable .meg files, followed by performing neighbour-joining phylogenetic analysis taking .meg files as inputs. The default Mega parameters were used when making NJ trees and performing bootstrap test (bootstrap replication time at 500).

Since Tar/TagB, Tar/TagF, TarL, and TarK share certain significant BLAST hits, we put them and their BLAST hits together to do ClustalW alignment and Mega analysis.

### MUMmer alignment and dot plot analysis

MUMmer package V3.0 was downloaded from TIGR ftp sites [16], which was used to align between the *B. subtilis *168 genome and each genome of the six *S. aureus *strains. Promer program was selected for its relatively high sensitivity. Promer generates amino acid alignments between two DNA input files which contain multiple sequences in FASTA format. Mummerplot was used to produce the dot-blot of the MUMmer alignments (Figure 2).

### Identification of full length *tarB *in *S. aureus *Mu50 and N315

The TarB orthologs identified from *S. aureus *Mu50 and N315 Refseq proteomes are remarkably shorter than TarB in *B. subtilis *W23, MRSA252, MSSA476 and COL, missing the N-terminal part and only about half the size of W23 TarB. We applied NCBI's ORF finder program [14] to analyze the TarB regions of N315 and Mu50. Two extended ORFs were identified which were similar to W23 TarB in length. Alignment analysis confirmed they are TarB orthologs in these two *S. aureus *strains (Figure 3).

The ORF in N315 for TarB locates in the genome from 687949 to 689052, and ORF in Mu50 for TarB from 712200 to 713303.

### The genomic organization of *tar *genes

The genomic localization information of Tar and Tag genes in *B. subtilis *168, W23 and *S. aureus *strains Mu50, MW2, N315, MRSA252, MSSA476, COL were retrieved from NCBI microbial genomes website [15]. Genomic organization maps of the five bacteria were then made (Figure 4, Additional file 4).

### Analysis of *tarIJL *duplication in *S. aureus *strains

BLASTP and phylogenetic analysis identified two copies of TarI, J and L in *S. aureus *Mu50, N315, MW2, MRSA252, MSSA476 and COL. From genomic analysis, TarI, TarJ and TarL in the analyzed *S. aureus *strains, are clustered into two *tarIJL *regions. To confirm that it is caused by gene duplication, we further performed alignment and dot-blot analysis of those two *tarIJL *regions in each of the *S. aureus *strains. [14]

We first cut out the relevant genomic sequences of *tarIJL *regions, including the intergenic regions among I, J, K genes and upstream 275 bp. Then we used dotmatcher program from EMBOSS package[17] to perform dot-blot analysis of the two *tarIJL *regions (Figure 5). We also ran NCBI bl2seq program to align the two regions (not shown).

## Authors' contributions

ZQ, YY and YZ did most of the analysis. LL helped with organizing the data. YJ identified the miss annotation of *S. aureus tar *genes in GenBank and proposed the analysis. YL and YJ supervised the analysis and interpreted the results. ZQ, LL and YJ read and approved the final manuscript.

## Supplementary Material

Additional File 1 **new TarB sequences.fasta**. New TarB sequences identified by NCBI ORFinder Click here for file

Additional File 2 **The *tar *and *tag *orthologs in S. aureus**. Identity and E-value are shown here. Newly identified TarB (named new_tarB) is also included. * Orthologs identified by dual-BLAST. ** Orthologs identified by Phylogenetic analysis (detail in figure 1). *** ORFs which the extension (new_tarB) are based. Click here for file

Additional File 3 **Annotations of *tar/tag *orthologs in NCBI**. NCBI annotations for tar/tag orthologs are shown here. Because of high homologous, tarA/tagA/tarB/tagB have been already annotated as teichoic acid biosynthesis protein, while other ORFs have not been well annotated. Click here for file

Additional File 4 **full size genomic organization image**. The top line of this figure is the divergon organization of tar and tag in *B. subtilis *W23 and 168 as a reference. The following six lines represented in arrows are graphic demonstration of genomic organization of the six *S. aureus *strains. Arrows in different colours represent different genes as illustrated by the colour table at the bottom. The length of each arrow is defined accurately by the scale, demonstrating the exact amino acid length of each gene. Figures below the arrows denote the GI numbers of corresponding gene. Figures between the arrows denote gap sizes in nucleotide unit between adjacent genes. Noting that, the black dots between some arrows denote the distances of corresponding genes, which are too long to illustrate by the normal scale. BLAST hits are also shown for each gene below. All six *S. aureus *strains share a similar genomic organization, which is quite different from their *B. subtilis *W23 and 168 counterparts. Click here for file

## Figures and Tables

**Table 1:** The BLAST homology between tar/tag genes and their orthologs in *S. aureus *strains. Newly identified TarB (named new_tarB) is also included. Orthologs of tagA/tarA are merged together. Same for tagD/tarD, tarF/tagF, tarB/tagB. Detail Blast Results are shown in Additional File 2 and annotations of these genes are shown in Additional file 3.

	N315	Mu50	MW2	COL	MSSA476	MRSA252
tagA	gi|15926313	gi|15923626	gi|21282327	gi|57651477	gi|49485508	gi|49482864
tarA						
tagD	gi|15926318	gi|15923631	gi|21282332	gi|57651482	gi|49485513	gi|49482869
tarD						
tagF	gi|5925967	gi|15923244	gi|21281959	gi|57652514	gi|49485134	gi|49482490
tarF						
tagB	New_tarB	New_tarB	gi|21282330	gi|57651480	gi|49485511	gi|49482867
tarB	*gi|15926316	*gi| 15926329				
tarI	gi|15925958	gi|15923245	gi|21281960	gi|57652515	gi|49485135	gi|49482491
	gi|15925954	gi|15923241	gi|21281956	gi|57652511	gi|49485129	gi|49482485
tarJ	gi|15925959	gi|15923246	gi|21281961	gi|57652516	gi|49485136	gi| 49482942
	gi|19525955	gi|15923242	gi|21281957	gi|57652512	gi|49485130	gi|49482486
tarL	gi|15925956	gi|15923243	gi|21281958	gi|57652513	gi|49485131	gi| 49482487
	gi|15925960	gi|15923247	gi|21281962	gi|57652517	gi|49485137	gi|49482493
tarK						

*ORFs which the extension (new_tarB) are based.

**Table 2:** Putative enzymatic functions of S. aureus Tar proteins

Gene	SwissProt
TarA	**N-acetylglucosaminyldiphosphoundecaprenol N-acetyl-beta-D- mannosaminyltransferase.**
TarB	**Putative CDP-glycerol:glycerophosphate glycerophosphotransferase tarB**
TarD	**Putative Glycerol-3-phosphate cytidylyltransferase**.
TarF	**Putative CDP-glycerol:glycerophosphate glycerophosphotransferase tarF**
TarI	**Putative D-ribitol-5-phosphate cytidylyltransferase**.
TarJ	**Putative ribitol-5-phosphate dehydrogenase**
TarK	**Putative ribitolphosphotransferase**
TarL	**Putative CDP-ribitol ribitolphosphotransferase**
