# Supplementary figures and images for: Genomic characterization of ribitol teichoic acid synthesis in Staphylococcus aureus: genes, genomic organization and gene duplication
Source: BMC Genomics. 2006 Apr 5;7:74. doi: 10.1186/1471-2164-7-74 (PMC1458327; doi:10.1186/1471-2164-7-74)

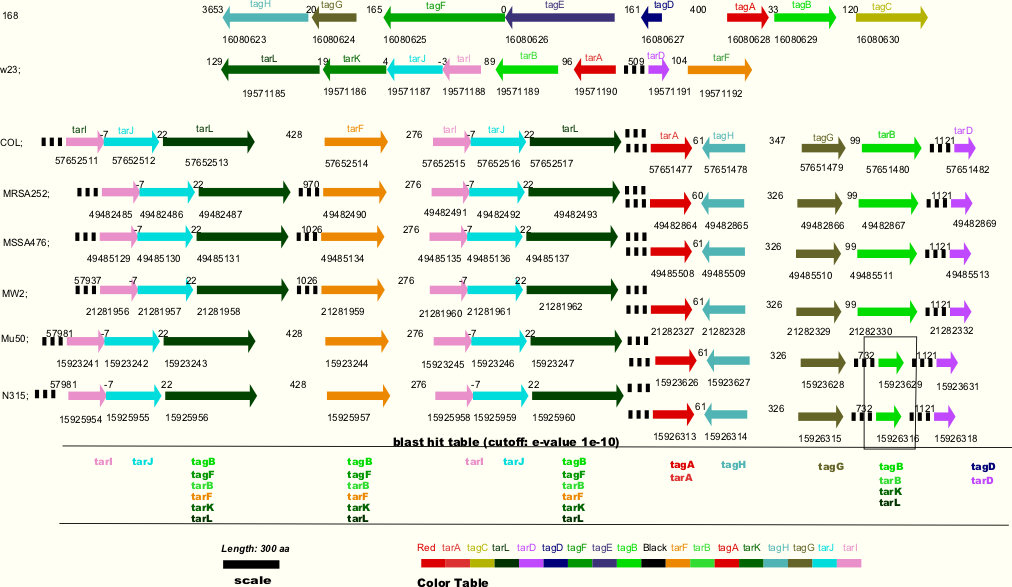

Supplement: Additional File 4 — full size genomic organization image. The top line of this figure is the divergon organization of tar and tag in B. subtilis W23 and 168 as a reference. The following six lines represented in arrows are graphic demonstration of genomic organization of the six S. aureus strains. Arrows in different colours represent different genes as illustrated by the colour table at the bottom. The length of each arrow is defined accurately by the scale, demonstrating the exact amino acid length of each gene. Figures below the arrows denote the GI numbers of corresponding gene. Figures between the arrows denote gap sizes in nucleotide unit between adjacent genes. Noting that, the black dots between some arrows denote the distances of corresponding genes, which are too long to illustrate by the normal scale. BLAST hits are also shown for each gene below. All six S. aureus strains share a similar genomic organization, which is quite different from their B. subtilis W23 and 168 counterparts. [file 1471-2164-7-74-S4.png]
